# The Effect of Pioglitazone and Metformin on Liver Function Tests, Insulin Resistance, and Liver Fat Content in Nonalcoholic Fatty Liver Disease: A Randomized Double Blinded Clinical Trial

**DOI:** 10.5812/hepatmon.9270

**Published:** 2013-05-21

**Authors:** Mohsen Razavizade, Raika Jamali, Abbas Arj, Seyyed Mohammad Matini, Alireza Moraveji, Effat Taherkhani

**Affiliations:** 1 Internal Medicine Ward, Shahid Beheshti Kashan Hospital, Kashan University of Medical Sciences, Kashan, IR Iran; 2 Research Development Center, Sina Hospital, Tehran University of Medical Sciences, Tehran, IR Iran; 3 Students Scientific Research Center, Tehran University of Medical Sciences, Tehran, IR Iran; 4 Department of Community Medicine, Kashan University of Medical Sciences, Kashan, IR Iran

**Keywords:** Fatty Liver, Insulin Resistance, Metformin, Pioglitazone

## Abstract

**Background:**

Non-alcoholic fatty liver disease (NAFLD) is considered as the hepatic manifestation of insulin resistance (IR) syndrome. The effect of insulin sensitizers on liver function tests and metabolic indices in NAFLD patients is a matter of debate.

**Objectives:**

The aim of study was to compare the effects of two different insulin sensitizers, pioglitazone, and metformin, on liver function tests (LFT), lipid profile, homeostasis model assessment-IR (HOMA-IR) index, and liver fat content (LFC) in NAFLD patients.

**Materials and Methods:**

This double blind clinical trial was performed on patients who were referred to a gastroenterology clinic with evidence of fatty liver in ultrasonography. After excluding other causes, participants with persistent elevated alanine aminotransferase (ALT) levels and “NAFLD liver fat score” greater than -0.64 were presumed to have NAFLD and were enrolled. They were randomly assigned to take metformin (1 g/day) or pioglitazone (30 mg/day) for four months. Fasting serum glucose (FSG), ALT, aspartate aminotransferase (AST), alkaline phosphatase (ALP), triglyceride, cholesterol (CHOL), high and low density lipoprotein (HDL, LDL), HOMA-IR, and LFC were checked at the baseline, two and four months post-treatment. LFC was measured by a validated formula.

**Results:**

Eighty patients (68 males) with mean age of 35.27 (± 7.98) were included. After 2 months, LFT was improved significantly in the pioglitazone group and did not change in the metformin group. After four months, both medications significantly decreased serum levels of LFT, FSG, CHOL, LDL, HOMA-IR, and LFC, and increased serum level of HDL. No statistically significant differences were seen between the two treatment groups with regard to the changes of laboratory parameters and LFC from baseline to four months post-treatment.

**Conclusions:**

During the four months, the use of metformin (1 g/day) and pioglitazone (30 mg/day) were safe and might have equally affected LFT, HOMA-IR, lipid profile, and LFC in NAFLD patients.

## 1. Background

Non-alcoholic fatty liver disease (NAFLD) is a common cause of chronic hepatitis that can lead to cirrhosis and hepatocellular carcinoma (1, 2). It is already considered as the hepatic manifestation of insulin resistance (metabolic) syndrome (3-5). The prevalence of NAFLD is rising worldwide due to the epidemic of obesity (6-8). Weight loss and obtaining an ideal body weight is a documented treatment option for NAFLD patients who are obese or overweight (9). However, the effects of pharmacological therapies in improvement of liver function tests and metabolic features of NAFLD patients need further investigations. Insulin resistance seems to predispose lipid accumulation within the liver and progresses to fibrosis in NAFLD (10). At present, prescription of insulin sensitizing drugs (like pioglitazone and metformin) has come to interest as a source to decrease insulin resistance in NAFLD patients. The improvement of glycemic control, lipid profile, and insulin sensitivity following the prescription of these medications in diabetic patients are well established. However, their effects on improvement of liver function tests, lipid profile, and insulin resistance in NAFLD patients remain controversial. Pioglitazone is a peroxisome proliferator activated receptor (PPAR)-gamma agonist that reduces insulin resistance in liver, muscle, and adipose tissue (11). The possible mechanism responsible for insulin sensitivity by pioglitazone is primarily promoting fatty acid uptake to adipose tissue that eventually results in decreasing serum fatty acids (11). This process is regulated by adiponectin (ADP) in adipose tissue (12). The ability of Pioglitazone to rise serum levels of ADP and to reduce serum levels of aminotransferase has been demonstrated by several trials (13-18). Weight gain and fat redistribution from the central area to the lower body was the most common side effect of this medication (13, 15, 17). Moreover, withdrawn due to hepatotoxicity was reported following pioglitazone treatment (14). Metformin is a biguanide drug that improves insulin sensitivity in the liver and skeletal muscle (19). Antihyperglycemic effect of metformin is mainly due to decreased gluconeogenesis and a slight effect on glycogenolysis (20). Several pilot trials reported that administration of Metformin resulted in reduced insulin resistance and improved aminotransferase levels without weight gain in NAFLD patients (21-23). On the other hand, another study showed no improvement in aminotransferase levels after administration of metformin (24). Review of the literature showed controversial results with regards to the effects of insulin sensitizing medications on liver function tests, and lipid profile in NAFLD patients.

## 2. Objectives

This randomized double blind clinical trial was designed to compare the effects of pioglitazone and metformin on liver function tests, lipid profile, HOMA-IR index, and liver fat content (LFC) in a sample of NAFLD patients.

## 3. Materials and Methods

### 3.1. Ethical Considerations

This study was carried out according to the ethical standards for human experimentation (Helsinki Declaration). The purpose of the study was explained to the participants and an informed written consent was taken.

### 3.2. Liver Ultrasonography

In this study, the radiologists compared the echogenicity of the right lobe of the and right kidney (in the sagittal view) for the detection of fatty liver. The existence of fat in liver parenchyma scatters the beam of ultrasound more than a non-fatty liver; therefore, the fatty liver appears hyperechogenic (25). Although ultrasonography might have some limitations for the grading of NAFLD, its availability makes it an appropriate tool for NAFLD screening (26). The radiologist was not informed about the clinical and laboratory data.

### 3.3. Subjects

Patients more than 18 years old with evidence of fatty liver in ultrasonography were enrolled in this study. These patients were referred to the gastroenterology clinic of a general hospital from January 2011 to January 2012 (Step 1). Patients with evidence of the following criteria were excluded from the study: alcohol use (more than 20 gram per day in men and 10 gram per day in women), type 1 diabetes mellitus, heart disease (ischemic or congestive), hepatic disease (viral hepatitis, autoimmune hepatitis, wilson disease, hemochromatosis, liver mass lesion), renal disease (serum creatinine concentration of > 1.5 mg/dl), any severe systemic co-morbidities, neoplasm, using any medication during the past 3 months, previous treatment (with thiazolidinediones, biguanides, or insulin), and pregnant or lactating women (Step 2). The serum aminotransferase levels ≥ 40 U/L were considered elevated (27). All participants with elevated serum aminotransferase levels in the first blood sample were rechecked in one month (lead-in phase). Participants with elevated aminotransferase levels in the second assessment were considered as having persistent elevated serum aminotransferase levels. This group of participants were presumed to have NAFLD and were included in the study if their “NAFLD liver fat score” value was greater than -0.64 (Step 3) (7).

### 3.4. NAFLD Liver fat Score and Liver fat Content Calculation

Performing liver biopsy for the determination of NAFLD has some limitations. Many patients due to its invasiveness, cost, and possible complications do not accept this method. We used “NAFLD liver fat score” instead of liver biopsy to determine NAFLD in our study (28). This score was calculated as below:

“NAFLD liver fat score = (- 2.89) + 1.18 * metabolic syndrome (yes = 1 / no = 0) + 0.45 * type 2 diabetes (yes = 2 / no = 0) + 0.15 * fasting serum insulin (mU/L) + 0.04 * fasting serum AST (U/L) – 0.94 * (AST/ALT)”.

The values greater than - 0.64 had a sensitivity of 86% and specificity of 71% for the prediction of NAFLD.

We used the formula that applied to the same variables for the measurement of LFC, as below: 

“Liver fat content (%) = 10 (-0.805 + 0.282 * metabolic syndrome (yes = 1 / no = 0) + 0.078 * type 2 diabetes (yes =2 / no =0) + 0.525 * log fasting serum insulin (mU/L) + 0.521 * log fasting serum AST (U/L) – 0.454 * log (AST/ALT)”.

The previous study had showed the validity of this equation for the prediction of LFC considering proton magnetic resonance spectroscopy (PMRS) as the gold standard (28). There was a correlation between LFC identified by PMRS and LFC calculated by the above formula (r = 0.7, P < 0.0001) (28).

### 3.5. Study Design

This study was designed as a double blind randomized controlled clinical trial (IRCT.IR ID: IRCT201105026361N1). During the four-month run in period, a dietitian interviewed the participants and recommended that they should not alter the daily calorie content of their diets.

### 3.6. Outcome Measures

The primary outcome measure was improvement in the serum aminotransferase concentration from baseline to the end of treatment at four months. The secondary outcome measures were changes in other biochemical parameters (liver function tests, lipid profile, and HOMA-IR) and LFC from baseline to the end of treatment at four months.

### 3.7. Sample Size Calculation

A statistical power analysis was utilized to determine the sample size. Based on a 50% response to pioglitazone according to the previous study, and power calculation (α = 0.8, β = 0.05), a total sample size of 66 patients was determined to detect one U/L inter-group difference in serum aminotransferase concentration (16). To allow for a possible 20% dropout rate, 80 patients (40 in each treatment group) were recruited in this double blind randomized clinical trial.

### 3.8. Randomization

According to a predefined computer-generated block randomization table with a 1:1 allocation, each of the patients was assigned to pioglitazone or metformin treatment groups. A random allocation sequence was generated by a clinical epidemiologist who was not aware of the treatment modalities using a computer software program (Microsoft Office Excel 2007, Microsoft Corp, Redmond, WA, USA). An investigator who was not involved in data collection and treatment, performed the enrollment of patients and their assignments into treatment groups.

### 3.9. Intervention

Lifestyle modification was the basis of treatment in this study and was provided to all participants. It consisted of providing a calorie-restricted diet to obtain an ideal body weight. The protocol used for diet in our study was based on “Guidelines for the diagnosis and management of nonalcoholic fatty liver disease: update 2010” (9). Rapid weight loss was avoided since it could deteriorate serum aminotransferase levels. A dietitian who was blinded to the study protocol checked the participants and controlled their daily calorie intake during the run in period. Eighty patients were randomized to receive either 30 mg/day of pioglitazone or 1 g/day of metformin for four months. To minimize the gastrointestinal side effects, metformin was taken at a dose of 500 mg/day. If the patients tolerated metformin, the dose increased gradually to one gram per day. A study coordinator that was not aware of patient’s data gave the medication to them every month in a sealed envelope (double blind design). The participants were requested to bring back the empty bottles of medications at the follow up visits every month. Compliance (by means of pill count) and adverse effects were checked at follow up visits.

### 3.10. Study Measurements

Height (meter) and weight (kilogram) of the participants were measured and body mass index (BMI) was calculated. Obese subjects were defined if their BMI was equal or greater than 30 kg/m^2^. Laboratory parameters were assessed at the general hospital at baseline, two and four months during the study period. After an overnight fast, sera of the participants were tested for fasting serum glucose (FSG), alanine aminotransferase (ALT), aspartate aminotransferase (AST), alkaline phosphatase (ALP), triglyceride (TG), cholesterol (CHOL), low-density lipoprotein (LDL) and high density lipoprotein (HDL) by enzymatic methods using Erba Mannheim auto analyzer XL-640 (Erba Diagnostics Mannheim, Germany). ALT, AST, and ALP levels were reported as unit per liter (U/L) and FSG, TG, CHOL, LDL and HDL levels were reported as milligrams per deciliter (mg/dl). Serum insulin concentration was measured using a commercially available kit (Biovendor, Brno, Czech Republic). The kit for determination of insulin (IR-insulin) in serum was based on a sandwich enzyme immunoassay. The procedure was performed according to the manufacturer’s manual, as follows. 96-well plate was coated with guinea pig anti human insulin antibody and insulin standard or samples were added to the wells for their immunoreactions. After incubation and plate washing, biotinylated guinea pig anti human insulin antibody was introduced to the wells and the antibody - antigen - labeled antibody complex was formed on the surface of the well. After rinsing out the excessive labeled antibody, HRP labeled streptavidin (SA-HRP) were added to bind to the labeled antibody. Finally, HRP enzyme activity was determined by o-Phenylenediamine dihydrochloride (OPD) and the concentration of insulin was calculated. Quantitative measurement of insulin resistance (IR) was performed using homeostasis model assessment-IR (HOMA-IR = fasting serum insulin x fasting serum glucose/22.5) (29).

To minimize the laboratory errors, the whole assay was performed by the same operator from the beginning to the end, and room temperature, air humidity, incubator temperature were strictly controlled. All the measurements were performed in duplicate. The intra-assay and inter-assay coefficient variations were less than 10% and 12% respectively.

### 3.11. Statistical Analysis

Data was summarized as means ± SD for continuous variables. The Kolmogorov-Smirnov test was used to evaluate the normal distribution of the continuous variables. Two-sample t-test was used to compare the mean values of continuous variables (laboratory, LFC, and anthropometric) between the metformin and pioglitazone groups. The chi-square test was used to compare categorical variables between the treatment groups. The changes in laboratory and LFC mean values between baseline, 2 and 4 months study period in each treatment group were calculated by paired t-test. The differences in laboratory and LFC mean value changes between (metformin and pioglitazone) groups were tested by two-sample t-test. Non-parametric methods were used for non-normally distributed values. The statistical analyses were performed using SPSS version 17 (SPSS, Chicago, IL, USA). The probability of the difference between the dependent and independent variables were considered significant if a two-tailed P value was less than 0.05.

## 4. Results

### 4.1. Patients Enrolled

Between January 2011 to January 2012, 93 patients suspected of having NAFLD were evaluated. Eighty patients with mean age of 35.27 (± 7.98) years were enrolled in the study. Reasons for exclusion were patient’s unwillingness to participate in the study (n = 7), normalization of ALT during the lead-in phase (n = 4), renal failure (n = 1), and pregnancy (n = 1). The frequency of participants with diabetes mellitus was six and twelve participants had impaired fasting glucose.

### 4.2. Medication Adverse Effects and Compliance

Significant side effects that need a decrease in the dose or discontinuation of the medications did not occur during the study period. Pill counts during the follow up visits discovered a good adherence to therapy with a mean consumption of 89% of expected tablets (range from 84 to 95).

### 4.3. Study Findings

Serum AST, ALT, ALP, FSG, TG, CHOL, and LDL concentrations were normally distributed at the baseline. (Z score = 1.6, 1.15, 1.23, 1.57, 1.7, 0.55, and 0.76 respectively, all P values > 0.05) Patients’ characteristics in treatment groups are shown in Table 1. The mean values of serum AST, ALT, ALP, TG, CHOL, LDL, HDL, FSG, HOMA-IR, and LFC at baseline, two and four months post-treatment according to treatment groups are provided in Table 2. The mean age, weight, BMI, laboratory values, LFC, and gender were similar between the treatment groups at baseline and during the study period. The comparisons of body weight, laboratory parameters, and LFC within the treatment groups along the study period are shown in Table 3. Mean body weight, serum AST, ALT, ALP, FSG, CHOL, LDL, HOMA-IR values, and LFC were significantly higher, but mean serum HDL level was lower at baseline than four months post-treatment in both treatment groups. The comparison of mean body weight, laboratory parameters, and LFC changes from baseline to two and four months post-treatment, and from two to four months post-treatment according to the treatment groups are provided in Table 4. The changes in mean body weight, laboratory values, and LFC from baseline to four months post-treatment were not significantly different between the treatment groups.

**Table 1.** Patients Characteristics According to the Treatment Groups

	Metformin	Pioglitazone	P value
**Age, y^a^, Mean ± SD **	36.35 ± 8.96	34.20 ± 6.79	0.23
**Gender, No.**			
Male	31	37	0.11
Female	9	3	0.50
**Obese, No.**	9	10	0.67
**Diabetes mellitus, No.**	4	2	0.15
**Weight at baseline, Kg^a^, Mean ± SD**	80.32 ± 5.92	83.47 ± 12.28	0.06
**Weight at 2 months, Kg^a^, Mean ± SD**	78.67 ± 6.73	83.08 ± 12.72	0.06
**Weight at 4 months, Kg^a^, Mean ± SD**	77.59 ± 7.90	82.30 ± 13.05	0.53
**Body mass index at baseline, Kg/m^2^^a^, Mean ± SD**	27.93 ± 2.28	27.48 ± 3.91	0.97
**Body mass index at 2 months, Kg/m^2^^a^, Mean ± SD**	27.32 ± 1.98	27.34 ± 3.99	0.84
**Body mass index at 4 months, Kg/m^2^^a^, Mean ± SD**	26.92 ± 2.18	27.06 ± 3.92	0.23

^a^The values of age, weight, and body mass index are expressed as mean ± standard deviation

**Table 2.** Mean Values (Standard Deviation) for Laboratory Parameters and Liver fat Content at Baseline, 2 and 4 Months Post-Treatment According to Treatment Group

	Metformin, Mean ± SD	Pioglitazone, Mean ± SD	Pioglitazone, Mean ± SD
**Aspartate Aminotransferase, U/L**			
**Baseline**	49.13 ± 16.81	50.47 ± 21.83	50.47 ± 21.83
**2 months**	47.37 ± 33.77	39.86 ± 11.27	39.86 ± 11.27
**4 months**	38.30 ± 19.88	36.72 ± 16.77	36.72 ± 16.77
**Alanine Aminotransferase, U/L**			
**Baseline**	87.72 ± 44.92	91.10 ± 38.17	91.10 ± 38.17
**2 months**	80.75 ± 66.44	67.59 ± 28.49	67.59 ± 28.49
**4 months**	65.97 ± 56.84	53.57 ± 26.03	53.57 ± 26.03
**Alkaline Phosphatase, U/L**			
**Baseline**	167.77 ± 30.85	163.42 ± 38.73	163.42 ± 38.73
**2 months**	169.60 ± 36.19	152.35 ± 38.94	152.35 ± 38.94
**4 months**	177.30 ± 47.38	152.00 ± 39.80	152.00 ± 39.80
**Triglyceride, mg/dl**			
**Baseline**	152.82 ± 74.81	137.82 ± 90.59	137.82 ± 90.59
**2 months**	173.95 ± 113.66	134.14 ± 57.68	134.14 ± 57.68
**4 months**	148.05 ± 52.95	134.66 ± 53.05	134.66 ± 53.05
**Cholesterol, mg/dl**			
**Baseline**	177.71 ± 33.96	178.58 ± 26.55	178.58 ± 26.55
**2 months**	183.92 ± 27.42	175.86 ± 27.23	175.86 ± 27.23
**4 months**	191.45 ± 31.83	180.20 ± 29.71	180.20 ± 29.71
**Low Density Lipoprotein, mg/dl**			
**Baseline**	95.45 ± 31.64	101.30 ± 18.72	101.30 ± 18.72
**2 months**	97.83 ± 29.37	97.38 ± 20.90	97.38 ± 20.90
**4 months**	109.52 ± 27.17	99.91 ± 22.91	99.91 ± 22.91
**High Density Lipoprotein, mg/dl**			
**Baseline**	51.41 ± 14.76	49.33 ± 10.30	49.33 ± 10.30
**2 months**	54.03 ± 16.03	51.02 ± 10.52	51.02 ± 10.52
**4 months**	52.00 ± 9.32	52.96 ± 8.83	52.96 ± 8.83
**Fasting Plasma Glucose, mg/dl**			
**Baseline**	94.52 ± 18.89	97.40 ± 12.92	97.40 ± 12.92
**2 months**	92.30 ± 16.52	91.30 ± 9.51	91.30 ± 9.51
**4 months**	95.47 ± 14.15	90.55 ± 11.21	90.55 ± 11.21
**HOMA-IR^a^**			
**Baseline**	3.14 ± 2.04	3.72 ± 4.82	3.72 ± 4.82
**4 months**	2.57 ± 1.47	2.45 ± 1.32	2.45 ± 1.32
**Liver Fat Content, %**			
**Baseline**	8.65 ± 5.11	9.75 ± 8.07	9.75 ± 8.07
**4 months**	6.12 ± 3.77	6.52 ± 4.12	6.52 ± 4.12

^a^Abbreviations: HOMA-IR: homeostasis model assessment-insulin resistance

**Table 3.** Intra-Group Comparisons of Mean Body Weight, Laboratory Parameters and Liver fat ContentDuring the Study Period

Treatment groups	Baseline vs. 2 months, P value	Baseline vs. 4 months, P value	2 months vs. 4 months, P value
**Body Weight, kg**			
**Pioglitazone**	0.42	0.04	< 0.01
**Metformin**	< 0.01	< 0.01	< 0.01
**Aspartate Aminotransferase, U/L**			
**Pioglitazone**	< 0.01	< 0.01	0.32
**Metformin**	0.67	< 0.01	< 0.01
**Alanine Aminotransferase, U/L**			
**Pioglitazone**	< 0.01	< 0.01	< 0.01
**Metformin**	0.31	< 0.01	< 0.01
**Alkaline Phosphatase, U/L**			
**Pioglitazone**	< 0.01	< 0.01	0.84
**Metformin**	0.09	< 0.01	< 0.01
**Triglyceride, mg/dl**			
**Pioglitazone**	0.75	0.77	0.94
**Metformin**	0.26	0.70	0.13
**Cholesterol, mg/dl**			
**Pioglitazone**	0.26	< 0.01	0.01
**Metformin**	0.26	< 0.01	< 0.01
**Low Density Lipoprotein, mg/dl**			
**Pioglitazone**	0.05	< 0.01	0.17
**Metformin**	0.69	< 0.01	0.10
**High Density Lipoprotein, mg/dl**			
**Pioglitazone**	0.27	< 0.01	< 0.01
**Metformin**	0.01	< 0.01	0.01
**Fasting Plasma Glucose, mg/dl**			
**Pioglitazone**	< 0.01	< 0.01	0.63
**Metformin**	< 0.01	< 0.01	0.26
**HOMA-IR^a^**			
**Pioglitazone**		< 0.01	
**Metformin**		< 0.01	
**Liver Fat Content, %**			
**Pioglitazone**		< 0.01	
**Metformin**		< 0.01	

^a^Abbreviations: HOMA-IR: homeostasis model assessment-insulin resistance

**Table 4.** Comparison of Body Weight, Laboratory Parameters and Liver fat Content Changes (mean ± SD) From Baseline to 2 and 4 Months Post Treatment, and From 2 to 4 Months Post Treatment According to the Treatment Groups

	Changes From Baseline to 2 Months			Changes From Baseline to 4 Months			Changes From 2 to 4 Months		
**Body weight, kg**	0.39 ± 2.98	1.65 ± 2.15	0.03	1.18 ± 3.70	2.73 ± 3.27	0.05	0.79 ± 1.76	1.08 ± 1.77	0.46
**Aspartate aminotransferase, U/L**	10.67 ± 17.23	1.75 ± 25.95	0.07	13.74 ± 27.1	10.82 ± 17.06	0.56	3.14 ± 20.01	2.22 ± 11.82	0.14
**Alanine aminotransferase, U/L**	23.51 ± 22.84	6.97 ± 11.82	0.03	37.52 ±40.70	21.75 ± 38.30	0.07	14.01 ± 31.95	2.22 ± 11.82	0.89
**Alkaline phosphatase, U/L**	11.07± 13.97	2.92 ± 10.94	0.00	11.42 ± 13.59	7.82 ± 6.36	0.13	0.35 ± 11.01	4.9± 7.95	0.04
**Triglyceride, mg/dl**	3.6 ± 7.63	-21.12 ± 11.82	0.26	3.16 ± 68.56	4.77 ± 77.94	0.92	-5.20 ± 43.24	25.9 ± 16.31	0.14
**Cholesterol, mg/dl**	2.71 ± 15.27	-6.21 ± 24.91	0.14	8.77 ± 8.70	11.08 ± 13.83	0.37	-4.28 ± 14.36	-7.5 ± 16.41	0.34
**Low density lipoprotein, mg/dl**	3.92 ± 12.54	-2.38 ± 18.65	0.33	6.52 ± 10.48	7.75 ± 9.93	0.58	2.6 ± 11.83	10.13 ± 38.52	0.24
**High density lipoprotein, mg/dl**	-1.68 ± 9.63	-2.62 ± 6.24	0.60	-7.08 ± 6.89	-4.98 ± 6.07	0.15	-5.39 ± 7.22	-2.36 ± 5.85	0.07
**Fasting plasma glucose, mg/dl**	4.92 ± 9.22	2.82 ± 8.94	0.30	5.35 ± 5.32	3.95 ± 4.23	0.19	0.42 ± 7.39	1.12 ± 7.35	0.67
**HOMA-IR^a^**	- b	-	-	0.76 ± 0.79	0.94 ± 0.58	0.26	-	-	-
**Liver fat content, %**	-	-	-	3.22 ± 4.95	2.53 ± 3.57	0.48	-	-	-

^a^Abbreviations: HOMA-IR: Homeostasis model assessment-insulin resistance

^b^Negative values represent the increase of the parameter at that interval

## 5. Discussion

The results of the present study showed that both treatment modalities were effective in reduction of serum levels of AST and ALT. Previous pilot trials demonstrated that metformin (1-1.5 g/day) was effective for reduction of ALT levels (21 - 23). In a twelve-month clinical trial in fifty-five NAFLD patients, it was shown that metformin (2 g/day) was better than a prescriptive weight reducing diet for reduction of ALT levels (30). In a 24-month pilot study on sixty children with NAFLD, Nobili et al. reported that administration of metformin (1.5 g/day) was effective for reduction of aminotransferase levels (31). However, metformin was not more effective than lifestyle intervention in reducing aminotransferase levels. In a pilot open label trial on fifteen NAFLD patients, Nair et al showed that metformin (20 mg/kg) reduced ALT and HOMA-IR in the first three months of study (24). However, after three months, there was no further decrease in insulin resistance and a rebound increase in ALT was observed. Improvement of AST and ALT following thiazolidinediones was reported in the previous trials. The results of a one year open-label study on sixty-three NAFLD patients showed that administration of rosiglitazone (4 mg/day for the first month and 8 mg/day thereafter) was more effective than placebo in reduction of aminotransferase level (32). Promrat et al. investigated the effect of pioglitazone (30 mg/day) on eighteen NAFLD patients for forty-eight weeks (13). The mean ALT level was significantly lower at the end of study (40 ± 25 U/L) compared to the baseline value (99 ± 71 U/L) (P <0.001). Sanyal et al. evaluated the ALT level in twenty non-diabetic NAFLD patients who were assigned to vitamin E (400 IU/ day) alone or the combination of vitamin E (400 IU/ day) and pioglitazone (30 mg/day) (14). In this six-month pilot study, serum ALT levels were normalized for all patients of both treatment groups. Lutchman et al. evaluated the effect of pioglitazone (30 mg/day) in twenty-one NAFLD patients for forty-eight weeks (15). After forty-eight weeks of pioglitazone therapy, the mean serum ALT level was significantly lower than the baseline values (75.7 ± 34.7 vs. 34 ± 12.7 U/L; P < 0.001). Forty-eight weeks after discontinuation of pioglitazone, the mean serum ALT level significantly increased (69.5 ± 38.7 U/L). This study concluded that in NAFLD patients, long-term administration of pioglitazone might be necessary to maintain the obtained results following treatment. In the study of Aithal et al., seventy-four non-diabetic NAFLD patients were assigned to take either pioglitazone (30 mg/day) or placebo for one year (16). In the pioglitazone group, the mean serum ALT level was significantly lower at the end of the study compared to the baseline value (55.9 ± 25.7 vs. 93.6 ± 61.3 U/L; P < 0.001). A study on fifty-five patients with NAFLD and impaired glucose tolerance or type two diabetes mellitus showed that pioglitazone (45 mg/day) was more effective than the placebo in reduction of serum ALT levels after six months (17). Bajaj et al studied the effect of pioglitazone (45 mg/day) in fourteen patients with type 2 diabetes mellitus (33). After sixteen weeks, the mean ALT level was significantly lower than the baseline value (22 ± 2 vs. 28 ± 3 U/L; P = 0.02). Tiikkainen et al. compared the effects of rosiglitazone (8 mg/day) and metformin (2 g/day) on serum ALT levels in twenty type 2 diabetic patients (34). After four months, serum ALT level decreased in the rosiglitazone group but remained unchanged in the metformin group. The effect of insulin sensitizers on the improvement of serum aminotransferase levels seems encouraging in the previous studies. The existence of controversy in their effect on serum aminotransferase levels and their sustained effect after discontinuation are the major drawbacks that need complementary investigations. We suggest that the differences in the results of the above mentioned trials might be due to the variations in the studies duration, patient’s heterogeneity (considering weight, gender, daily physical activity, and the level of IR), and the medication doses. In this study, the serum ALP was decreased significantly from baseline to four months post-treatment in both treatment groups. An improvement of serum ALP level was reported following pioglitazone treatment (13 , 14). These observations are in concordance with our study. However, there was an increase in serum ALP level following metformin treatment in the study by Nair et al. (24). It should be noted that total amounts of ALP cannot be considered as a specific marker for liver disease. The hepatic isoenzyme is a more reliable marker for the detection of liver disease. Therefore, the rise of total serum ALP following administration of metformin in the above-mentioned study might be related to conditions unrelated to NAFLD. Documentation of this observation with concomitant checking of ALP hepatic isoenzyme or gamma glutamyl transpeptidase seems a reasonable approach. In this study, the serum metabolic parameters (FSG, HOMA-IR, CHOL, LDL, and HDL) and LFC were improved significantly from baseline to four months post-treatment in both treatment groups. These findings are in accordance with the previous trials (13 - 17 , 21 - 24). In the present study, serum TG level did not change significantly in both treatment groups during the study period. This finding is in accordance with the results of previous trials that reported serum TG level did not change following pioglitazone prescription in NAFLD patients (13 , 16 , 17). The results of previous trials on the effect of metformin on serum TG level in NAFLD patients are somewhat controversial. Magalotti et al. studied the effect of metformin (1.5 g/day) in eleven NAFLD patients for six months (21). The mean serum TG level did not change significantly from baseline (176 ± 104 mg/dl) to the end of treatment (142 ± 61 mg/dl). Marchesini et al. studied the effect of metformin (1.5 g/day) in twenty NAFLD patients for four months (23). The mean serum TG level did not change significantly from baseline (1.91 ± 1.12 mmol/L) to the end of treatment (1.92 ± 1.06 mmol/L) in the metformin group. In the study of Nair et al, serum TG level did not change significantly following a twelve month administration of metformin for NAFLD patients (24). Nobili et al. showed that metformin (1.5 g/day) reduced serum TG levels in NAFLD patients after 12 months (31). The differences in the results of the above-mentioned trials might be due to the variations in the studies duration, patients’ heterogeneity (considering weight, gender, daily physical activity, and the level of IR), medication doses, and possible concomitant familial hyperlipidemia. To the best of our knowledge, this is the first clinical trial on NAFLD patients that compared the effect of pioglitazone and metformin on liver function tests (including AST, ALT, and ALP), metabolic profile (including FSG, HOMA-IR, and lipid profile), and LFC. No statistically significant differences were seen between the treatment groups with regard to the changes of laboratory parameters and LFC from baseline to four months post-treatment. It seems that metformin and pioglitazone might equally affect the liver function tests, HOMA-IR, CHOL, LDL, HDL, and LFC in NAFLD patients. Weight gain after pioglitazone treatment was reported in previous studies (13 , 15 - 17). In this study, there was no significant weight change in the participants receiving pioglitazone during the study period. Our finding was similar to that of Sanyal et al. that reported BMI did not increase significantly in subjects receiving pioglitazone (14). Although the daily calorie intake of the participants was checked by a dietitian during the follow up visits, but daily physical activity was not monitored in this study. This shortcoming might explain the absence of weight gain in the pioglitazone group of our study. Obtaining an ideal body weight is the mainstay of treatment in NAFLD. Hypocaloric diet and administration of appropriate daily physical activity help the NAFLD patient reduce their body weight. In this study, a dietitian supervised participants’ daily calorie intake. The dietitian was blinded to the treatment groups and controlled daily calorie intake in follow up visits. The protocol used for diet in our study was based on “Guidelines for the diagnosis and management of nonalcoholic fatty liver disease: update 2010” (9). Hypo caloric diets resulted in weight reduction in both treatment groups from baseline to the end of study (Table 3). Meanwhile, body weight reduction was not statistically different between treatment groups (Table 4). The above findings show that hypocaloric diets resulted in a significant reduction of body weight in participants in each treatment groups; Moreover, body weight reduction that was due to the hypocaloric diet, was not statistically different in treatment groups. Therefore, it can be concluded that diet was not different in treatment groups and might not have biased the results. We should note the role of metformin in NAFLD based on the recent guideline (2012) from the American Association for the Study of Liver Diseases, American College of Gastroenterology, and the American Gastroenterological Association. It has been mentioned that metformin does not have significant effects on liver histology. It is not recommended as a specific treatment for liver disease in adults with NAFLD (35). It is obvious that our study was designed and conducted (in 2011) before the release of this guideline.

### 5.1. The Limitations of the Study

Serum aminotransferase levels seem to have fluctuations in the natural course of disease (even without any therapy) in NAFLD patients (36). Therefore, the lack of control group was a limitation of study. Serum ALT value has limitations in predicting NAFLD (37). Although the participants were presumed to have NASH according to the study protocol, the diagnosis of NASH was not confirmed by liver histology. Therefore, the absence of liver biopsy was another limitation of this study in documenting NASH patients. Exercise and weight reduction are the factors that influence the treatment of NAFLD. The lack of control on participants’ daily exercise in this study might be considered as another limitation. The duration of study period was rather short, the sample size was small, and the sustained effects of these medications were not evaluated after discontinuation of treatment.

### 5.2. Recommendation for Future Studies

Since pioglitazone and metformin reduce the IR by different mechanisms, combined therapy with these drugs may show more improvement in aminotransferase concentrations than either of them alone. Comparing the effects of combination therapy with pioglitazone and metformin versus pioglitazone alone on histologic and biochemical changes in NAFLD patients with longer follow up duration together with well-established control groups is recommended.

### 5.3. Summary

This double blind randomized clinical trial was performed on eighty NAFLD patients who were randomly assigned to metformin and pioglitazone treatments. Administration of either medication significantly led to a decrease in the serum AST, ALT, ALP, FSG, CHOL, LDL, HOMA-IR, and LFC, and an increase in the serum HDL. No statistically significant differences were observed between the treatment groups with regards to changes of laboratory parameters and LFC from baseline to four months post-treatment. It seems that metformin and pioglitazone might equally affect liver function tests, HOMA-IR, CHOL, LDL, HDL, and LFC in NAFLD patients. These medications could be suggested as a safe treatment option for the management of NAFLD patients.
